# Quantitative Prediction of the Location of Carotid Bifurcation and Neurovascular Structures in the Carotid Region: A Cross-Sectional Cadaveric Study

**DOI:** 10.1155/2021/9214104

**Published:** 2021-11-28

**Authors:** Daniel W. Griepp, Abin Sajan, Robert DiRaimo, Lev Starikov, Samuel Márquez

**Affiliations:** ^1^Department of Surgery, SUNY Downstate Health Sciences University, Brooklyn, NY, USA; ^2^Department of Cell Biology, SUNY Downstate Health Sciences University, Brooklyn, NY, USA; ^3^Department of Radiology, Columbia University Irving Medical Center, New York, NY, USA; ^4^BlueRock Therapeutics, New York, NY, USA; ^5^Department of Otolaryngology, SUNY Downstate Health Sciences University, Brooklyn, NY, USA

## Abstract

**Introduction:**

The carotid region is encountered in vascular and neurological surgery and carries a potential for vascular and cranial nerve trauma. The carotid bifurcation is an especially important landmark and difficult to predict based on currently established landmarks. This study is a detailed analysis of the carotid region and proposes a novel methodology to predict the height of the bifurcation.

**Materials and Methods:**

Superficial and deep dissections were performed on the anterior triangle of the neck to expose the carotid region in twenty-one formalin-fixed donor cadavers. Musculoskeletal and neurovascular structures were assessed in relation to the carotid bifurcation and the medial border of the clavicle (MBC).

**Results:**

The carotid bifurcation occurred, on average, 11.4 mm higher on the left (*p* < 0.001; 95% CI: 9.28, 13.54). The superior thyroid artery (*p* < 0.001), facial vein (*p* < 0.001), and cranial nerve XII (*p* < 0.001) were all more distal on the left side when measured from the MBC while the angle of the mandible and stylohyoid muscle remained symmetric. Left- and right-sided vascular structures were symmetric when measured from the carotid bifurcation.

**Conclusions:**

Neurovascular structures within the carotid region are likely to be anatomically superior on the left side while vessels are likely to remain symmetric in relation to the carotid bifurcation. When measured from the MBC, the bifurcation height can be predicted by multiplying the distance between the MBC and mastoid process by 0.65 (right side) or 0.74 (left side). This novel methodological estimation may be easily learned and directly implemented in clinical practice.

## 1. Introduction

The anatomical structures of the carotid region are encountered in neurovascular procedures ranging from carotid endarterectomy (CEA), an open vascular procedure commonly performed for stroke prevention in patients with atherosclerotic plaque buildup in the internal carotid artery (ICA), to anterior cervical spine surgery, such as anterior discectomy and cervical fusion (ACDF), to treat spinal cord compression [1–3]. In CEA, considered a delicate surgery because of the small surgical field, intraoperative retraction and cross-clamping of carotid arteries leave cranial nerves and adjacent structures at risk for injury [4–8]. In ACDF, the carotid structures are often retracted laterally to expose the vertebral bodies, which increases the potential of damage to branches of the external carotid, such as the superior thyroid artery [9]. Thus, an in-depth understanding of the surgical anatomy as well as key landmarks of the carotid region is important to carefully navigate structures and ensure minimal intraoperative complications [8, 10–13].

The height of the carotid bifurcation is an important intraoperative landmark and is currently defined in the literature in relation to its anterior (hyoid bone and thyroid cartilage) and posterior (cervical vertebral levels C3-C4) landmarks [14–18]. These landmarks are generally not useful for procedures of the head and neck given they are not readily available and hard to estimate with a patient lying in the operative position. A recent study suggested that more investigation of the carotid region with the head extended and contralaterally turned would be more meaningful [16]. Moreover, differences between the left and right sides in relation to the carotid bifurcation are currently poorly characterized in the literature, which is important given both the right and left carotid regions are routinely surgically approached.

This study quantifies the anatomy of the carotid region, specifically focusing on neurovascular structures that surround the carotid bifurcation, and proposes a novel method to predict the height of the carotid bifurcation during preoperative markup.

## 2. Materials and Methods

Twenty-five adult Caucasian formalin-fixed donor bodies, housed in the collection of the State University of New York (SUNY) Downstate Medical Center anatomy laboratory cold room, were randomly selected and exclusively chosen for this study. Inspectional analysis of material showed all donors to be morphologically normal with no signs of trauma or congenital abnormalities. After the dissection process, a final sample size of twenty-one donor bodies was found suitable for the study.

The common carotid systems were dissected using an anterior triangle of the neck approach to ensure that the carotid bifurcations were clearly delineated. Parameters of the field of dissection were established from the medial border of the clavicle (MBC) to the mastoid process (MP). The specific bony landmark of the MBC was determined by measuring the absolute joint space of the sternoclavicular junction and dividing in the frontal plane by half to identify the midpoint of the MBC at its medial border. The MP end point was determined by locating and marking the dome of the most posterolateral prominence, posterior to the helix of the outer ear. All arterial branches of the carotid arteries, along with veins, muscles, and nerves contiguous to the carotid bifurcation, were dissected and identified on both sides of the neck region. All donors were kept in supine position with measurements made with the head laterally rotated 45 degrees away from the side of dissection with the neck in slight extension to mimic a patient in operative position.

**Figure 1:**
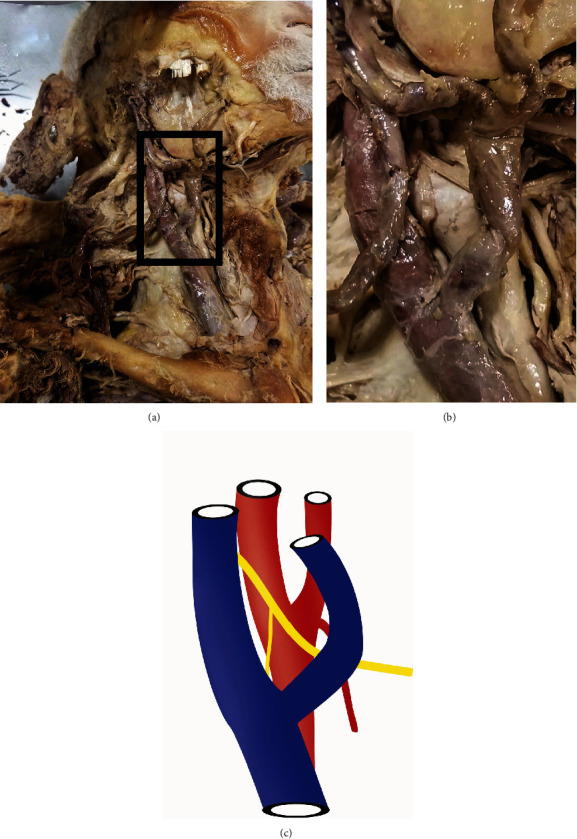
Superficial dissection of the carotid region. The anterolateral neck was dissected to reveal the jugular (a) and facial (b) vein. (c) The superficial relationships: internal jugular vein (blue), hypoglossal nerve with superior root of the ansa cervicalis, and common carotid artery (red) bifurcating into the internal and external. The vagus nerve is not visualized above.

**Figure 2:**
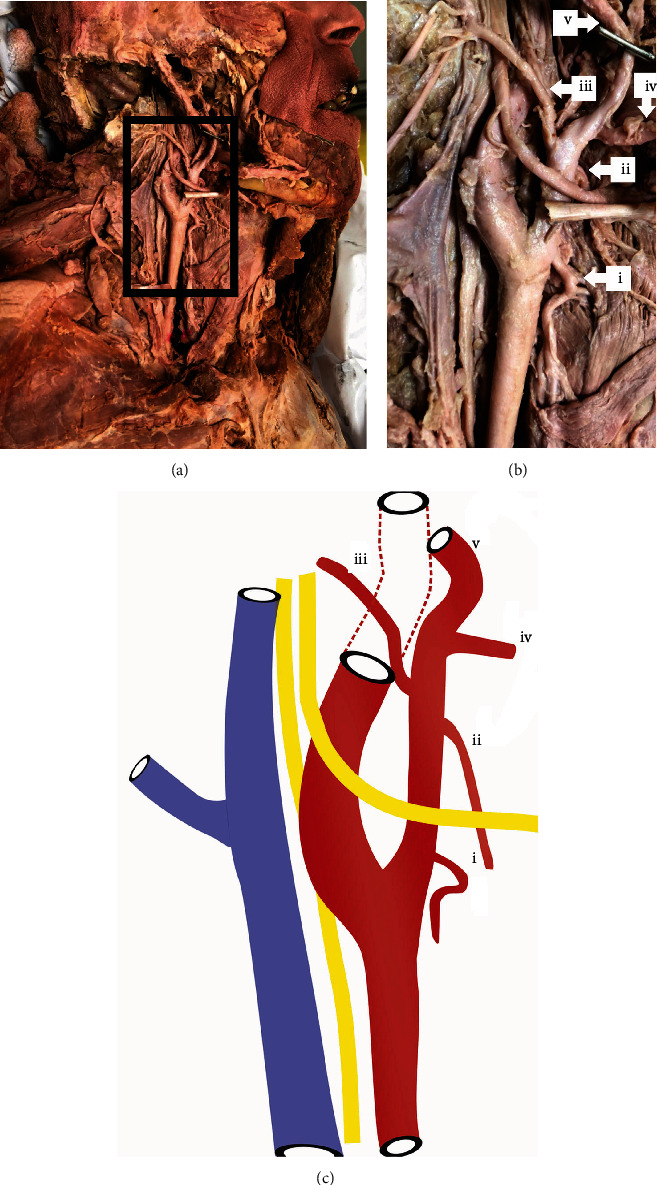
Deep dissection of the carotid region. The venous vessels (a) have been reflected laterally (b) to reveal the underlying the arteries. (c) Summary of the relationship with the internal jugular vein (blue), vagus nerve traveling inferiorly, hypoglossal nerve coursing laterally over the bifurcation, and the common carotid artery (red) with its internal and external branches. i: STA; ii: lingual artery; iii: occipital artery; iv: facial artery; v: external carotid artery. All branches of the external carotid artery have not been visualized. Note that the angle of the mandible was removed to maximize vessel branch visibility for this image.

**Figure 3:**
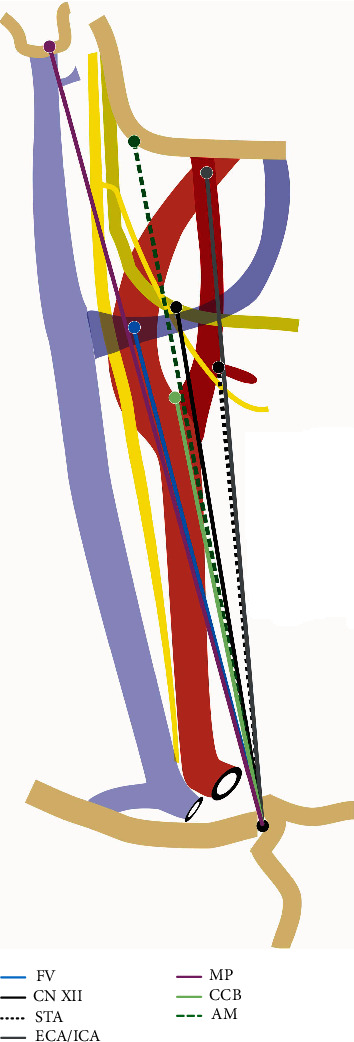
Right-sided animation demonstrating measurements from the medial border of the clavicle (MBC) to points of interest. Structures are drawn to best depict measurement trajectories and not necessarily to scale. The following structures are abbreviated: FV: facial vein; CN XII: hypoglossal nerve; STA: superior thyroid artery; ECA/ICA: point above the bifurcation where the external-internal carotid artery crossover occurs; MP: mastoid process; CCB: common carotid bifurcation; AM: angle of mandible.

**Figure 4:**
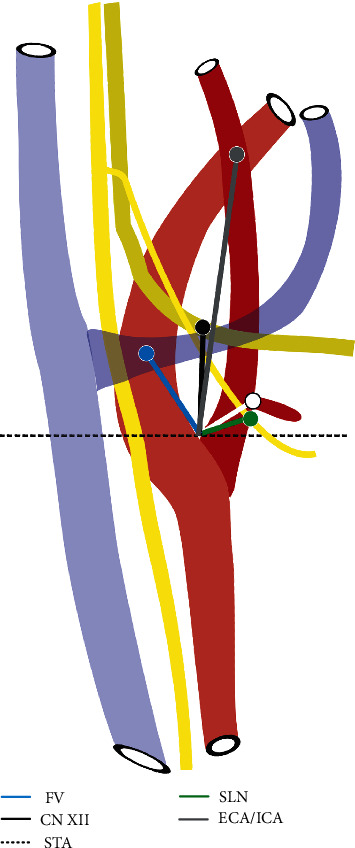
Right-sided animation demonstrating measurements from the carotid bifurcation to points of interest. The dotted line is drawn at the level of the carotid bifurcation. The following structures are abbreviated: FV: facial vein; CN XII: hypoglossal nerve; STA: superior thyroid artery; SLN: superior laryngeal nerve; ECA/ICA: point above the bifurcation where the external-internal carotid artery crossover occurs.

A Neiko 01409A electronic Vernier digital caliper obtained all measurements with accuracy to a hundredth of a millimeter. The dissection was divided into two sections with the superficial (Figure 1) and deep (Figure 2) layers. The following measurements for vasculature included MBC to carotid bifurcation, superior thyroid artery (STA), and facial vein (measured as the point where facial v. passes over the carotid artery). Additional measures were carotid bifurcation to facial vein, stylohyoid muscle insertion on hyoid bone, and external-internal carotid artery (ECA/ICA) crossover. Measurements for nervous structures included carotid bifurcation to hypoglossal nerve (CN XII) over internal carotid artery (ICA) and to superior laryngeal nerve (SLN). Further measurements included origin of STA to CN XII when it overlaps with ICA and SLN. The posteromedial location of the CN X was identified and confirmed on each side. The point selected as and reported as the carotid bifurcation was the most inferior point of the notch created by the ICA and ECA branch points, superior to the common carotid artery. The entire span of the studied region is depicted visually in Figure 3, and the orientation of measurements of the carotid region is shown in Figure 4.

Anonymized data were initially recorded in Microsoft Excel and then transferred to JASP Team (2020), JASP (Version 0.14.1), and GraphPad Prism 9.2.0 (283). Data were tested for normality and homogeneity of variance. Paired *t*-test was used to evaluate for differences in right-sided and left-sided structures. A *p* value of less than 0.05 was considered statistically significant.

## 3. Results

Of the twenty-five-adult formalin-fixed donors dissected, four were excluded from the final sample as structures of interest were damaged during the dissection process and could not be measured. All anatomical structures on both right and left sides were complete from the remaining dissected cadavers (*n* = 21), comprising a total of 42 carotid regions that provided the measurements analyzed in this study. Descriptive statistics were performed on all measurements to assess central tendency and variability to describe the characteristics of the data set (see Table 1).

The mean distance from the MBC to the carotid bifurcation was 109 mm (SD ± 11; range 88-135; 95% CI 104-114) overall, 104 mm (SD ± 10; range 88-120; 95% CI 99.8-108) on the right side, and 115 mm (SD ± 11; range 98-135; 95% CI 111-120) on the left. A paired *t*-test found the mean distance was 11.4 mm higher on the left (*p* < 0.001; 95% CI: 9.28, 13.54).

Additional paired analyses of all measurements were performed to identify differences in right versus left neurovascular structures in the carotid region of each individual cadaver in relation to the MBC (bony landmark) and the carotid bifurcation (vascular landmark) (Table 2). To account for size variability across cadavers, ratios of measurements were also generated by dividing measurements by the longest measured distance between landmarks of individual cadavers (MBC to MP) of the corresponding side (Table 3).

**Figure 5:**
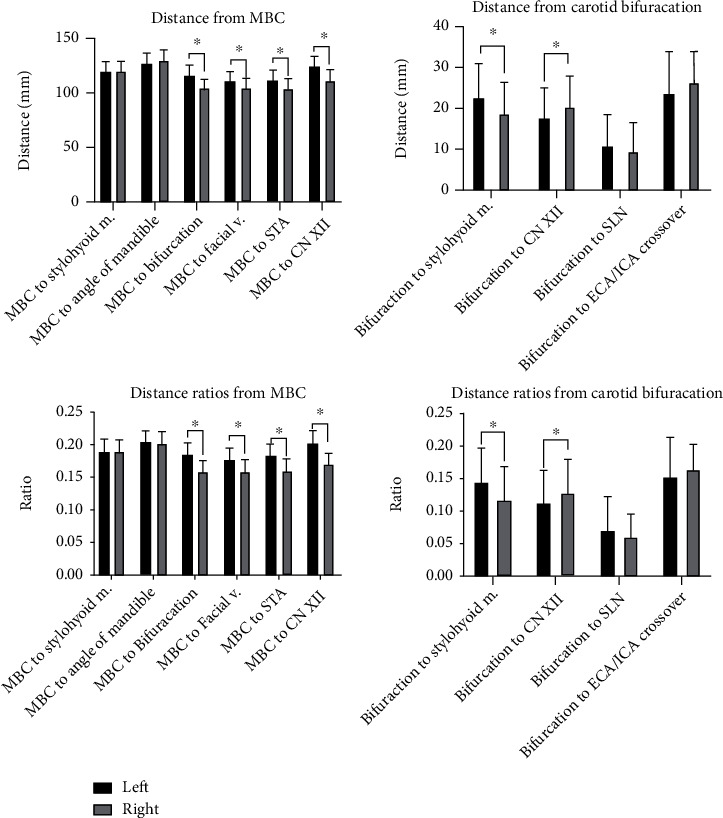
Distances and distance ratios of carotid structures, in relation to the medial border of the clavicle (MBC) and carotid bifurcation, are presented graphically with standard deviation (error bars). The following structures are abbreviated: STA: superior thyroid artery; CN XII: hypoglossal nerve; ECA/ICA: point above the bifurcation where the external-internal carotid artery crossover occurs. ^∗^denotes statistical significance of *p* < 0.001.

With respect to the MBC, there were no significant differences of right versus left measurements to bony landmarks (MP, angle of mandible) and muscles of the neck (i.e., stylohyoid muscle). Neurovascular structures were significantly higher on the left side, including the height of the carotid bifurcation (*p* < 0.001; SE 1.02; 95% CI 9.28-13.54), STA (*p* < 0.001; SE 1.31; 95% CI 5.80-11.26), facial vein (*p* < 0.001; SE 0.88; 95% CI 12.05-15.71), and cranial nerve XII (*p* < 0.001; SE 0.91; 95% CI 10.64-14.42) when measured from the MBC. With respect to the carotid bifurcation, significant differences were found from measurements to the stylohyoid muscle insertion on the left and right sides (*p* < 0.001; SE 0.74; 95% CI 2.82-5.89). Left- and right-sided vascular structures were not found to be statistically different in relation to the corresponding carotid bifurcation. The distance from the carotid bifurcation to the hypoglossal (cranial nerve XII) was significantly less on the left side when compared with the contralateral side (*p* = 0.003; SE 0.90; 95% CI -4.98 to -1.21). After data was normalized through generation of measurement ratios, all findings remained consistent with no change in level of statistical significance. Graphical comparison of measurements and ratios is depicted in Figure 5.

**Figure 6:**
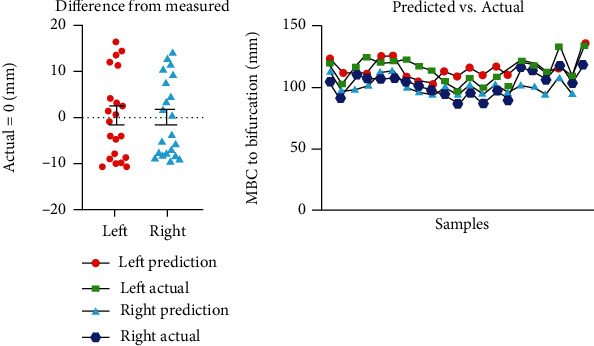
(a) Difference of actual versus predicted measurements with respective means (solid line) with standard error of the mean (error bars). (b) Absolute predicted versus measurements.

When measured from the MBC, the carotid bifurcation was found 0.65 away from the right-sided MBC and 0.74 from the left-sided MBC (in terms of ratio of total distance from the corresponding sided MBC to mastoid process) (see Table 3). These ratios were then tested by comparing true measured bifurcation heights to retrospectively generated predictions (using 0.65 or 0.74). Paired analysis of both left- and right-sided values revealed no statistically significant difference between true and predicted bifurcation heights (Table 4). Figurative representations of actual and predicted measures are shown in Figure 6.

## 4. Discussion

We report a detailed description of muscular, vascular, and nervous structures of the carotid region with anatomical structures maintained in the operative position. In our analysis, novel measurements of the carotid region from two different fixed points in the human anatomy were generated: the MBC (Figure 3) and the notch point of the carotid bifurcation (Figure 4). All nervous and vascular structures measured from the MBC landmark were significantly higher on the left side when compared with the right. Conversely, all muscular and bony landmarks, when measured from the carotid bifurcation, were found to be significantly different in right versus left sides. Interestingly, the only structure that was consistently asymmetric with respect to right or left sidedness and did not meaningfully correlate with any bony, muscular, or vascular anatomy was the hypoglossal nerve (CN XII). Our findings indicate that neurovascular structures of the head and neck positioned anatomically superior on the left side when compared to the contralateral side. When measuring from the novel MBC in our study, left- and right-sided anatomical symmetry was observed between bony landmarks and muscular structures, which indicates that significant differences identified in the nervous and vascular anatomy are true differences.

Previous studies measuring the height of the carotid bifurcation from different landmarks have generally found the left bifurcation to be higher, although the right side has also been reported as higher [15, 16, 19, 20]. In our series, the carotid bifurcation was significantly higher on the left side (mean difference 11.4 mm) with no single patient having a measured carotid bifurcation that was higher on the right side when compared with contralateral side. This difference is likely related to embryological development of the aortic arches and the branch point of the left common carotid artery from the brachiocephalic trunk, rather than the aorta [21].

An in-depth understanding of left versus right carotid regions and how they relate to one another is particularly relevant clinically given the carotid region may be approached surgically from either the right or left side. Surgeons may become more comfortable navigating a unilateral side of the neck, such as neurological surgeons who may prefer left-sided approaches when performing ACDF's. Thus, in cases when opposite sided approaches are required, surgeons should expect to find neurovascular structures more inferiorly placed on the right side, when compared to the contralateral side. Additionally, in cases where a patient has particularly difficult anatomy, such as a short neck, the knowledge that left-sided vascular structures are anatomically superior may affect preoperative planning and clinical decision-making, especially if endovascular versus open approaches are being considered [22].

The height of the carotid bifurcation, in relation to clinical landmarks, has been described to lie anteriorly between the superior borders of the thyroid cartilage and hyoid bone or posteriorly at the C3 or C4 intervertebral disc level [14–16]. Use of the vertebral body as a landmark would be limited to spine surgery when intraoperative fluoroscopy is readily available [13, 23]. Moreover, the hyoid bone and the thyroid cartilage is unreliable given it may be found from C3 to C6 intervertebral levels depending on degree of head extension [17]. Currently, there is no known way to predict the height of the carotid bifurcation if a surgeon wished to do so during marking of the surgical incision and/or anatomical sites on patient's skin, prior to surgical procedures.

This study proposes a method by which clinicians can identify, with a high degree of accuracy, the height of the carotid bifurcation. An estimation of the bifurcation point can be done by directly measuring from the MBC, in a superior-lateral trajectory, along the anterior border of the sternocleidomastoid muscle. In our sample, the bifurcation generally occurred 10.4 cm above the MBC on the right (25th-75th percentile: 9.8 to 10.9) and 11.5 cm above the MBC on the left (25th-75th percentile: 10.8 to 12.3). However, these measurements have limited applicability given the inherent variation found in donors and may lead to misleading estimations in patients outside of population norms.

The most accurate prediction of the carotid bifurcation is achieved through a two-step process: (1) measuring the distance from the MBC to the mastoid process and then (2) multiplying that number by either 0.65 for right-sided prediction or 0.74 for left-sided prediction (see Table 3). As seen in Table 4, the internal validity of this method was tested by comparing true measured bifurcation heights to retrospectively generated predictions using the present method. Paired analysis of both left- and right-sided values revealed no statistically significant difference between true and predicted bifurcation heights. All predictions (42/42) fell within 1.5 cm of the expected height, and 69% (29/42) of predictions were within <1 cm each actual value (Figure 6).

Although more samples in future studies could potentially generate a more robust model, inevitable anatomical variation would likely contribute to similar findings regarding variance in data. Indeed, the present analysis of the anatomical variation of the region allowed us to obtain actual measurements of these structures, rather than idealized estimations, which is of critical importance and more valuable in everyday practice [23, 24]. Both proximity of the MBC to the surgical field and the closeness of predictions to true values make the MBC a more useful clinical landmark to predict the location of the carotid bifurcation with the patient in operative position. It should be noted that the carotid vessels are deep structures, and while study measurements were performed on these dissected structures, measurements preoperatively would be performed superficially on the surface of the skin. The authors, nevertheless, find that the novel method proposed in this study is a clinically useful, straightforward, and easy method to predict the height of the carotid bifurcation. Moreover, the closeness of predicted heights to actual heights demonstrates that an acceptable error given surgical incision in this region is relatively larger than the range of error demonstrated in predictions (>1.5 cm above or below actual height). We encourage pursuance of future studies that can further assess validity through other methods, such as measuring on a different set of cadavers or with prospective studies on patients with noninvasive ultrasonic measurements to locate the carotid bifurcation.

## 5. Conclusion

This study offers a descriptive analysis of musculoskeletal, vascular, and nervous structures of the carotid region based on readily available surgical landmarks. When navigating the carotid region, results showed that neurovascular structures are likely to be found anatomically superior on the left side. In contrast, arterial and venous elements are likely to remain anatomically symmetric in relation to each corresponding carotid bifurcation, regardless of side. The hypoglossal nerve lies slightly, yet significantly, superior on the left side while more proximal to the left carotid bifurcation. The height of the carotid bifurcation may be reliably predicted by multiplying the distance between the MBC and mastoid process by either 0.65 on the right side or 0.74 on the left side and tracking the distance in a superolateral trajectory along the sternocleidomastoid muscle from the MBC. This novel method may be easily learned and directly implemented in clinical practice. The present study serves as an anatomical review of clinically relevant structures for surgeons and young surgeons in training who will be navigating this region and require intimate familiarity with subtle differences of the left- and right-sided carotid anatomy. This may ultimately aid during surgical dissection and potentially mitigate surgical complications, leading to better surgical outcomes.

## Figures and Tables

**Table 1:** Summary of measurements with statistical description.

Distance (*N* = 42)	Mean	SEM	Median	SD	Variance	Range (min–max)
MBC to mastoid process	157.81	1.59	155.76	10.79	116.44	51.14 (139.56–190.7)
MBC to angle of mandible	127.54	0.94	127.80	6.35	40.37	25.27 (116.45–141.72)
MBC to stylohyoid m.	117.52	1.50	116.18	10.15	102.92	37.45 (102.09–139.54)
MBC to carotid bifurcation	107.95	1.63	108.18	11.08	122.78	47.64 (87.56–135.2)
MBC to STA	105.18	1.54	107.58	10.43	108.83	38.84 (84.57–123.41)
MBC to facial vein	106.45	1.35	105.94	9.17	84.08	37.76 (91.25–129.01)
MBC to CN XII	117.43	1.45	118.08	9.84	96.86	38.67 (96.51–135.18)
Bifurcation to stylohyoid m.	21.73	1.39	22.48	9.47	89.70	38.53 (0.39–38.92)
Bifurcation to ECA/ICA	25.77	1.33	25.45	9.00	81.02	45.02 (0.98–46.00)
Bifurcation to facial vein	11.70	0.87	11	5.92	34.99	22.79 (0.03–22.82)
Bifurcation to CN XII	19.99	1.38	20.84	9.35	87.47	36.14 (3.16–39.3)
Bifurcation to SLN	10.09	0.99	10.07	6.71	45.02	25.31 (0.09–25.4)

MBC: medial border of the clavicle; SEM: standard error of mean; SD: standard deviation; STA: superior thyroid artery; CN XII: hypoglossal nerve; ECA/ICA: point above the bifurcation where the external-internal carotid artery crossover occurs; SLN: superior laryngeal nerve.

**Table 2:** Summary of left vs. right comparison of carotid measurements.

						95% CI for mean difference	
Distance (*n* = 21)	Left Mean ± SD (mm)	Right Mean ± SD (mm)	*p*	Mean difference	SE difference	Lower	Upper
MBC to angle of mandible	127.29 ± 7.94	129.18 ± 6.42	0.260	-1.89	1.63	-5.29	1.51
MBC to stylohyoid m.	118.28 ± 10.46	120.46 ± 11.33	0.170	-2.19	1.53	-5.38	1.01
MBC to carotid bifurcation	115.47 ± 10.69	104.06 ± 9.94	<.001	11.41	1.02	9.28	13.54
MBC to STA	111.35 ± 8.58	102.83 ± 12.26	<.001	8.53	1.31	5.80	11.26
MBC to facial vein	113.72 ± 7.59	99.84 ± 5.71	<.001	13.88	0.88	12.05	15.71
MBC to CN XII	124.58 ± 7.75	112.05 ± 9.72	<.001	12.53	0.91	10.64	14.42
Bifurcation to stylohyoid m.	22.59 ± 8.65	18.24 ± 8.80	<.001	4.36	0.74	2.82	5.89
Bifurcation to facial vein	−0.004 ± 12.26	0.68 ± 14.66	0.270	-0.68	0.60	-1.93	0.57
Bifurcation to ECA/ICA	23.69 ± 9.91	26.22 ± 7.91	0.162	-2.53	1.74	-6.15	1.10
Bifurcation to CN XII	17.32 ± 8.17	20.41 ± 8.17	0.003	-3.09	0.90	-4.98	-1.21
Bifurcation to SLN	10.65 ± 7.10	9.46 ± 6.34	0.475	1.18	1.63	-2.21	4.58

MBC: medial border of the clavicle; STA: superior thyroid artery; CN XII: hypoglossal nerve; ECA/ICA: point above the bifurcation where the external-internal carotid artery crossover occurs; SLN: superior laryngeal nerve.

**Table 3:** Summary of left vs. right comparison of normalized carotid ratios.

						95% CI for mean difference	
Normalized distance ratios^∗^ (*n* = 21)	Left Mean ± SD	Right Mean ± SD	*p*	Mean difference	SE difference	Lower	Upper
MBC to angle of mandible	0.818 ± 0.062	0.809 ± 0.056	0.571	-1.889	0.016	-0.024	0.042
MBC to stylohyoid m.	0.760 ± 0.081	0.753 ± 0.069	0.589	-2.185	0.014	-0.021	0.036
MBC to carotid bifurcation ‡	0.740 ± 0.062	0.650 ± 0.054	<.001	11.410	0.011	0.068	0.113
MBC to STA	0.715 ± 0.063	0.641 ± 0.056	<.001	8.529	0.006	0.062	0.087
MBC to facial vein	0.731 ± 0.062	0.625 ± 0.047	<.001	13.879	0.007	0.091	0.121
MBC to CN XII	0.802 ± 0.075	0.703 ± 0.080	<.001	12.528	0.008	0.082	0.115
Bifurcation to stylohyoid m.	0.143 ± 0.052	0.114 ± 0.056	<.001	4.356	0.005	0.019	0.039
Bifurcation to facial vein	0.001 ± 0.077	0.006 ± 0.090	0.178	-0.679	0.003	-0.011	0.002
Bifurcation to ECA/ICA	0.150 ± 0.061	0.162 ± 0.039	0.279	-2.527	0.010	-0.033	0.010
Bifurcation to CN XII	0.110 ± 0.050	0.125 ± 0.055	0.005	-3.091	0.005	-0.026	-0.005
Bifurcation to SLN	0.068 ± 0.045	0.058 ± 0.037	0.334	1.183	0.010	-0.011	0.032

^∗^MBC to mastoid process (MP) = 1; ratios normalized individually by cadaver specific distances. ‡ Used in final model to predict bifurcation height. MBC: medial border of the clavicle; STA: superior thyroid artery; CN XII: hypoglossal nerve; ECA/ICA: point above the bifurcation where the external-internal carotid artery crossover occurs; SLN: superior laryngeal nerve.

**Table 4:** Internal validity of true versus predicted bifurcation height using novel method.

						95% CI for mean difference	
Side (*n* = 21)	Prediction	True (measured)	*p*	Mean difference	SE difference	Lower	Upper
Right CCB	104.23 ± 7.61	104.06 ± 9.94	0.93	0.17	1.86	-3.71	4.04
Left CCB	115.56 ± 7.91	115.47 ± 10.69	0.97	0.09	2.05	-4.19	4.37

CCB: common carotid bifurcation.

## Data Availability

Original data from samples will be available upon request.
